# Surgical Intervention Outcome for Patients With Acute Vision Loss Due to Allergic Fungal Rhinosinusitis: A Systematic Review and Meta-Analysis

**DOI:** 10.7759/cureus.69886

**Published:** 2024-09-21

**Authors:** Abdulrahman A Otaif, Ammar A Najmi, Ibrahim M Shajry, Riyadh A Jahlan, Abdulrahman A Daghreeri, Abdulelah A Otaif, Hatoun A Alali, Ghadah Khalid H Alanazi, Wejdan A Al Mustafa, Deema F Alharbi, Lujain B Alotaibi, Amal S Althagafi, Shahad S Aladwan

**Affiliations:** 1 Faculty of Medicine, Jazan University, Jazan, SAU; 2 Department of Otorhinolaryngology - Head and Neck Surgery, Prince Mohammed bin Nasser Hospital, Jazan, SAU; 3 Department of Ophthalmology, Prince Mohammed bin Nasser Hospital, Jazan, SAU; 4 Faculty of Medicine, University of Tabuk, Tabuk, SAU; 5 General Practice, Northern Borders Health Cluster, Arar, SAU; 6 Department of Ophthalmology, Aljaber Hospital for Eyes and ENT, Al-Ahsa, SAU; 7 Department of Surgery, Aldiriyah Hospital, Riyadh, SAU; 8 Department of Ophthalmology, King Abdulaziz Specialist Hospital, Taif, SAU; 9 Faculty of Medicine, Qassim University, Buraydah, SAU

**Keywords:** acute vision loss, allergic fungal rhinosinusitis, meta-analysis, optic neuropathy, rhinosinusitis, systematic review

## Abstract

Allergic fungal rhinosinusitis (AFRS) is a severe type of chronic rhinosinusitis (CRS) characterized by a high risk of acute vision loss resulting from the affection of optic nerves. This vision loss results from direct pressure upon the optic nerve by the sinus tissues and bone degradation. This is why early involvement of a professional is significant so as not to result in a permanent disability. This systematic review and meta-analysis will analyze the efficacy of surgical operations in enhancing visual outcomes in patients with acute vision loss due to AFRS. A systematic search was carried out based on the Preferred Reporting Items for Systematic Reviews and Meta-Analyses (PRISMA) guidelines using PubMed, Embase, Cochrane Library, and Scopus with the keywords "acute vision loss," "allergic fungal rhinosinusitis," and "surgical intervention." The studies included patients who developed AFRS-related acute vision loss, those assessing the outcomes of the surgical interventions, and those published in English. Two separate researchers extracted data and conducted quality assessments. The data synthesis process employed the R studio software (Posit, Boston, MA). The identified studies for the review constituted 12 and involved 320 patients. The mean pooled event rate regarding acute vision loss of AFRS patients was 33.1%. Ear, nose, and throat surgeries, including functional endoscopic sinus surgery (FESS) and optic nerve decompression, had a mean pooled success in vision recovery of 68%. Early treatment and utilization of other types of corticosteroids were utilized to enhance the results. Hence, early and proper surgery like FESS, optic nerve decompression, and corticosteroid treatment have better visual prognosis in AFRS patients. Standard early diagnosis and management guarantee the prevention of irreversible blindness, which stresses the need for an interdisciplinary approach and further studies.

## Introduction and background

Allergic fungal rhinosinusitis (AFRS) is a type of CRS in which there is an enhanced immunologic reactivity to the common fungal elements in the environment [1,2]. This condition mostly affects immunocompetent people, and it is more prevalent in areas with warm and humid climates, where fungal spores are more common. Symptoms often characterize AFRS, such as nasal stuffiness, sinus headaches, and nasal polyps [3]. However, in the advanced stages, the following complications may arise orbital and intracranial involvement [4]. 

AFRS has some serious consequences, and one of the worst is acute vision loss, which develops when the disease process spreads to the orbit and affects the optic nerve [5]. The causes of vision loss in AFRS include direct pressure from the inflamed sinus tissues on the optic nerve, erosion of the bony sinus walls, exposure of the optic nerve, and presumably an autoimmune reaction that produces inflammation [6,7]. It is essential that the condition is diagnosed as early as possible and treatment commences since if the condition is left for a long time, the child may end up being blind [8]. 

In most cases, the standard management of AFRS consists of surgical and medical procedures [9]. FESS surgery is intended to remove the fungus burden and the characterized inflamed mucosa, correct sinus obstruction, and apply optic nerve decompression if required [10]. Since the condition is inflammatory, medical treatment is typically needed to reduce or prevent inflammation from returning [10]. Despite the above therapies, the management approach of AFRS-related acute vision loss is still unknown, and further research is needed to establish the different outcomes of the different surgical methods. 

This research is deemed necessary since AFRS-related acute vision loss can be disastrous and significantly affect the patient's quality of life. Blindness substantially limits the ability to perform activities of daily living and results in disability if appropriate treatment is not immediately provided. Surgeons and clinicians need to comprehend the results of operations to create a knowledge-based practice that can enhance the patient’s prognosis and protect against further vision loss.

Due to differences in surgical approaches and the absence of clear evidence about the most effective strategies, this systematic review and meta-analysis will offer a summarized assessment of the existing practices. This review attempts to determine from the data from different studies which surgical interventions provide the best results in patients with AFRS-associated acute vision loss. Moreover, this proposed research seeks to identify aspects, such as the timing of the surgery and the application of other complementary treatments, that determine the success of the surgery. 

Aim and objective

This systematic review and meta-analysis focus on assessing the surgical interventions that enhance the visual acuity of patients diagnosed with acute vision loss due to AFRS.

## Review

Methodology

Search Strategy

This systematic review and meta-analyses were reported based on the Preferred Reporting Items for Systematic Reviews and Meta-Analyses (PRISMA) statement. A precise approach was adopted in the literature review to contain information on surgical intervention outcomes for vision-impaired patients with AFRS. The search was performed in PubMed, Embase, Cochrane Library, and Scopus databases. The search terms were meticulously chosen to cover all aspects of the topic. They included combinations of the following keywords: "acute vision loss," "allergic fungal rhinosinusitis," "surgical intervention," "endoscopic sinus surgery," "optic nerve decompression," "FESS," "visual outcomes," and "AFRS.” Boolean operators (AND, OR) were used to comprehend the search and not miss anything. The literature review covered a 25-year period from 1999 to 2024.

Inclusion and Exclusion Criteria

The study's inclusion criteria were studies involving patients with acute vision loss due to AFRS, studies evaluating the outcomes of surgical interventions, peer-reviewed articles published in English, and prospective, retrospective, case series, and case reports. The exclusion criteria included studies not involving surgical intervention, studies without specific data on visual outcomes, non-peer-reviewed articles, editorials, commentaries, and articles not available in English.

Study Screening

The preliminary search generated a large number of articles, which were then filtered. The titles and abstracts of all identified articles were screened to exclude papers that did not qualify for inclusion in the review by two authors. Inter-reviewer disagreements were handled through consultation and agreement. Studies identified in the previous step were retrieved in full text for further evaluation of whether they met the inclusion criteria.

Data Extraction

Two researchers manually extracted data using a data extraction form with the same content. Data extraction involved study characteristics such as authors, publication year, study design, and details of the patient's characteristics, as well as details of the surgical intervention, follow-up, and specific vision loss outcomes. The main focus of the study was the change in the incidence of acute vision loss after surgery. Secondary outcomes also concern the rates of recurrences and the general prognosis of the patients.

Data Synthesis

For the metanalysis, the R studio software (Posit, Boston, MA) was used to synthesize data. The primary outcome data were summarized with the random-effects models to control for the variation across the studies. Heterogeneity was tested using the I^2^ statistic, where a value exceeding 50% was deemed to show significant heterogeneity. Exploratory subgroup analyses were done according to the type of surgical procedure and the preoperative duration of the vision loss. Sensitivity analyses were also conducted to examine the stability of the results obtained. The findings were reported as the overall standardized mean difference (SMD) with a 95% CI for each outcome.

Results

**Figure 1:**
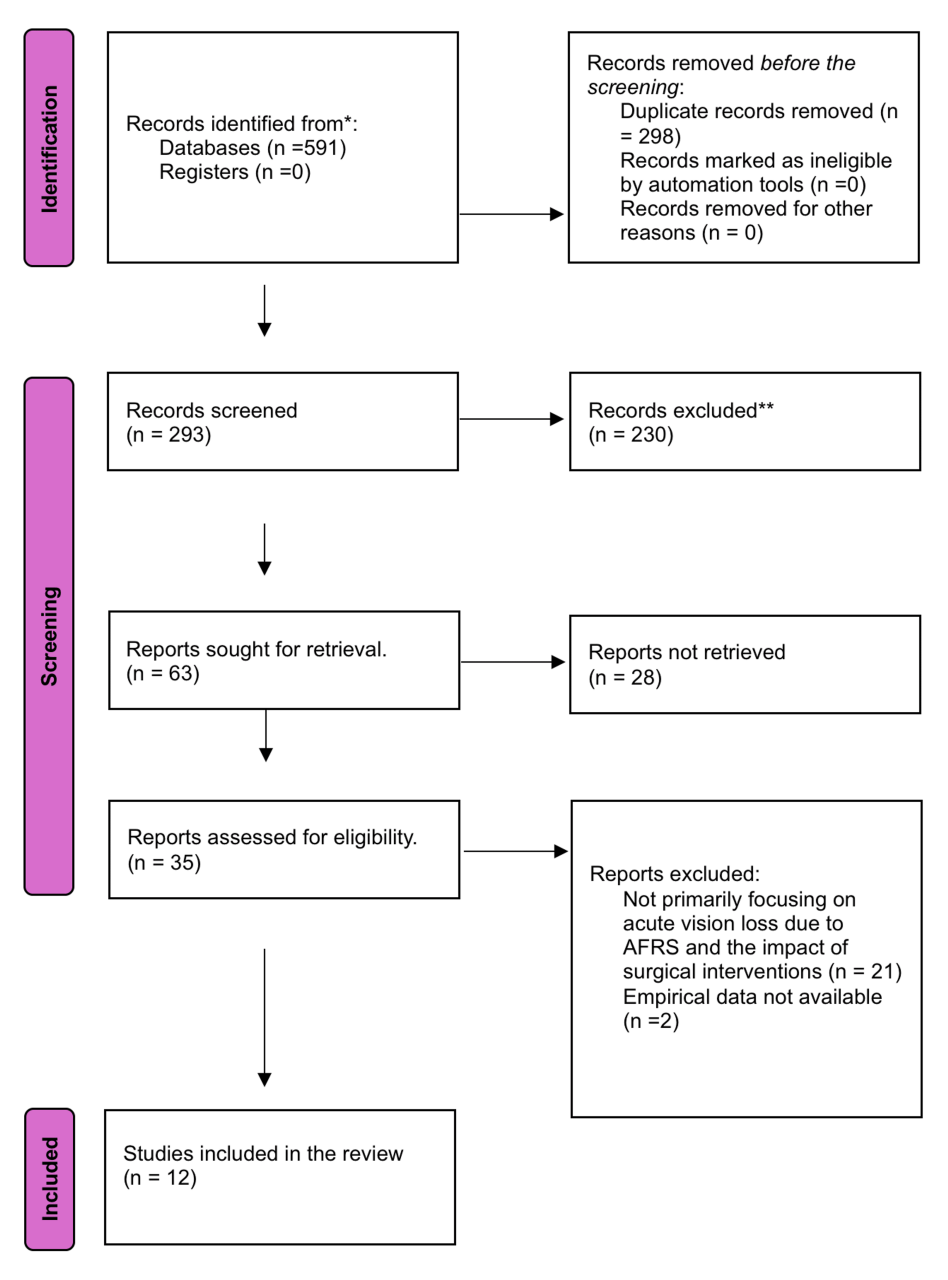
Preferred Reporting Items for Systematic Reviews and Meta-Analyses (PRISMA) flow diagram of included studies

The search through various databases generated 591 papers. After excluding duplicates and applying the inclusion and exclusion criteria, this systematic review and meta-analysis included 12 studies to determine the prognosis of patients who underwent surgery for acute vision loss resulting from AFRS. Figure 1 represents the detailed PRISMA flow diagram of the included studies' selection and inclusion process. The included studies reflected a range of study designs, including prospective and retrospective studies, case series, and case reports to identify a broad spectrum of evidence on the different surgical interventions for managing vision impairment due to AFRS. The detailed characteristics of the included studies are given in Table 1.

**Table 1:** Study characteristics of included studies

Study title	Authors and reference citation	Study type	Aim and objective	Problem	Patients involved	Surgical intervention used	Follow-up period	Positive impact on acute vision loss	Reason of vision loss	No impact	Negative impact	Recurrence rate	Overall outcome	Impact of surgical intervention to reverse vision loss
Ophthalmic manifestations of allergic fungal sinusitis	Carter et al. [11]	Case series	To highlight allergic fungal sinusitis as a cause of ophthalmic and sinus problems and present a successful treatment approach	Acute vision loss due to fungal rhinosinusitis	Six patients	The combined approach between otolaryngology and ophthalmology, including anterior and posterior ethmoidectomies, sphenoidotomy, bilateral frontal sinus trephination, and orbital roof exploration	Mean follow-up of 34 months	One out of six patients had severe but reversible visual loss.	Compression of the optic nerve, possibly fungal toxic effects	Five patients	None reported permanent visual loss	No recurrence	Successful treatment with no complications and no recurrence of symptoms at follow-up.	Vision loss was reversed in the patient who had severe visual loss following surgical intervention and corticosteroid therapy.
Management of fungal rhinosinusitis: experience from a tertiary care centre in North India	Monga et al. [12]	Retrospective	To review the types, clinical presentation, microbiology, histopathology, and outcomes related to fungal rhinosinusitis (FRS)	Acute vision loss due to fungal rhinosinusitis	30 patients	Functional endoscopic sinus surgery (FESS), optic nerve decompression, endoscopic debridement	Mean follow-up of 21.4 months	Immediate recovery of visual acuity in the patient with the fungal ball following optic nerve decompression; both AIFRS patients with visual loss were disease-free at one year of follow-up.	Optic nerve compression due to fungal ball, mucormycosis invasion in AIFRS	Not specified	Not specified	16.6% in non-invasive FRS, 20.8% in invasive FRS	Favorable outcomes with timely surgical and medical management; nil mortality at one year of follow-up.	Successful reversal of vision loss in the patient with a fungal ball; effective disease management in AIFRS patients with vision loss.
Acute versus chronic invasive fungal rhinosinusitis: a case-control study	Piromchai and Thanaviratananich [13]	Case-control study	To determine clinical presentation, complication, and morbidity in patients with acute versus chronic invasive fungal rhinosinusitis (IFS)	Acute vision loss due to fungal rhinosinusitis	59 patients	Endoscopic debridement or external approach, antifungal therapy with Amphotericin B	Average hospital stay of 30.58 ± 26.43 days	Out of 22 patients with acute vision loss, 15 showed improvement post-surgery.	Mucosal necrosis, black crust/debris indicative of acute IFS	Seven patients	14 patients in the acute IFS group died (31.11% mortality rate); no deaths in the chronic IFS group.	Not specified	Acute IFS was found more commonly in immunocompromised patients with higher morbidity and mortality; chronic IFS patients had a better survival rate.	Surgical intervention showed a 68% success rate in reversing acute vision loss among affected patients.
Orbital complication of allergic fungal rhino-sinusitis	Alzarei and Assiri [14]	Retrospective	To determine the standard features of orbital complications due to allergic fungal rhino-sinusitis (AFRS)	Acute vision loss due to fungal rhinosinusitis	60 patients	Endoscopic debridement, surgical, and medical treatment	Not specified	Out of nine patients with unilateral complete blindness, six showed improvement post-surgery.	Direct or indirect optic nerve compression, inflammatory process resulting in optic neuritis	Three patients	Not specified	Not specified	AFRS can present with severe orbital complications, including eye proptosis, diplopia, and unilateral complete blindness. Early diagnosis and treatment are crucial to prevent serious complications.	Surgical intervention showed a 67% success rate in reversing unilateral complete blindness among affected patients.
Optic nerve compression in allergic fungal sinusitis	Thakar et al. [15]	Prospective	To describe the syndrome of optic nerve involvement in cases of allergic fungal sinusitis	Acute vision loss due to fungal rhinosinusitis	70 patients (10 with optic nerve involvement)	Urgent functional endoscopic sinus surgery or external sphenoethmoidectomy, optic nerve decompression	Regular follow-ups at one, three, and six months, and one year postoperatively	Visual recovery in seven out of 13 eyes (54%).	Optic nerve compression due to bony erosion of the lateral sphenoid wall	Six out of 13 eyes	None specified	Not specified	Early surgical decompression can reverse visual loss in cases with recent onset; prolonged visual loss and complete loss are poor prognostic factors.	Surgical intervention showed a 54% success rate in reversing vision loss among affected eyes.
Acute vision loss in patients with allergic fungal rhinosinusitis: a case series	Allami et al. [16]	Case series	To present and analyze cases of acute vision loss in patients with AFRS and assess the outcomes of surgical intervention	Acute vision loss due to fungal rhinosinusitis	Three patients	Endoscopic sinus surgery with orbital decompression	Over 12 months	No meaningful improvement in vision was observed in any patient despite successful nerve decompression.	Compression of the optic nerve due to extensive nasal polyps and bone erosion in the bilateral orbits and lateral wall of the sphenoid sinus	Three patients	None specified	Not specified	Despite surgical and medical management, no improvement in vision was observed in the cases presented. Early intervention is crucial to prevent complications.	Surgical intervention did not reverse vision loss in the presented cases.
Outcomes of pressure-induced cranial neuropathies from allergic fungal rhinosinusitis	Illing et al. [17]	Retrospective	To describe strategies for AFRS-induced neuropathies and evaluate ophthalmologic outcomes following endoscopic sinus surgery	Acute vision loss due to fungal rhinosinusitis	Nine patients	Endoscopic surgical decompression of the sinuses and oral steroid therapy	9 to 13 days, four to six weeks, three months, and six-month intervals	Seven patients had a complete return of nerve function; two had partial recovery.	Compression of the optic nerve or abducens nerve due to bone erosion of the orbital walls or cranial base	Not specified	Two patients had partial recovery with early and abrupt improvement within two weeks of surgery.	Not specified	Prompt surgical decompression and removal of disease, along with aggressive medical therapy, provided excellent outcomes.	The surgical intervention showed a high success rate, with 82% of affected eyes achieving full recovery from visual compromise following endoscopic removal of the disease combined with postoperative medical management.
Loss of vision outcome for allergic fungal sinusitis: case report and literature review	Alhussien et al. [18]	Case report and literature review	To identify factors associated with visual outcomes in cases of acute vision loss due to AFRS	Acute vision loss due to fungal rhinosinusitis	50 patients	FESS, corticosteroid therapy	Two years	17 cases had complete recovery, and 10 cases had partial recovery.	Optic nerve compression due to fungal mass and inflammation	14 cases	None specified	High, continued surveillance recommended	Early diagnosis and intervention can return vision to normal; delayed presentation and complete vision loss are associated with worse outcomes.	The surgical intervention showed complete recovery in 34% of cases and partial recovery in 20%.
Extensive allergic fungal rhinosinusitis: ophthalmic and skull base complications	Vashishth [19]	Retrospective	To review the clinical features, ophthalmic and skull base complications, radiologic correlates, surgical methods, and outcomes in cases of extensive AFRS	Acute vision loss due to fungal rhinosinusitis	11 patients	Endoscopic sinus surgery with complete disease clearance	Minimum one year, maximum three years	Complete visual recovery in the patient with sudden vision loss; partial or no recovery in those with prolonged vision loss	Optic nerve compression due to erosion of the optic canal by fungal debris and diseased mucosa	Three patients	Not specified	One case of disease recurrence 18 months post-surgery	Early intervention with surgical decompression and steroids can lead to better visual outcomes. Long-term follow-up is critical to manage recurrences and monitor sinus patency.	Surgical intervention showed complete recovery in cases of sudden vision loss and partial/no recovery in cases of prolonged vision loss.
Allergic fungal rhinosinusitis involving frontal sinus: a prospective study comparing surgical modalities	Gupta et al. [20]	Prospective	To compare the postoperative results of endoscopic frontal sinusotomy with external frontoethmoidectomy approach in patients with AFRS involving the frontal sinus	Acute vision loss due to fungal rhinosinusitis	40 patients (16 in Group I, 24 in Group II)	Group I: external frontoethmoidectomy; Group II: endoscopic endonasal frontoethmoidectomy	Six months to 2.5 years (mean follow-up of 11 months)	Five out of eight patients with acute vision loss showed improvement post-surgery.	Bony erosion with sinus infection causing optic nerve compression	Three patients	Not specified	Group I: 4.76% (1 out of 21 sinuses); Group II: 8.18% (3 out of 37 sinuses)	Both surgical modalities showed high success rates, with no significant difference between the groups. After six months of follow-up, Group I had a success rate of 95.5%, while Group II had a success rate of 91.1%.	Surgical intervention showed a 62.5% success rate in reversing acute vision loss among affected patients.
Management of allergic fungal rhinosinusitis associated with vision loss during COVID-19 era	Singh et al. [21]	Case series	To present cases of AFRS-associated vision loss managed during the COVID-19 pandemic and to discuss the impact of delayed treatment.	Acute vision loss due to fungal rhinosinusitis	Four patients (six eyes with vision loss)	FESS to remove the disease and decompress the optic nerve	Immediate to two months postoperative follow-up	Vision recovery in four out of six eyes (66.7%).	Optic nerve compression due to pressure from AFRS-related inflammation and fungal debris	Two eyes	None specified	Not specified	Timely surgical intervention generally results in favorable outcomes. Delays in diagnosis and treatment can lead to irreversible vision loss.	Surgical intervention successfully reverses vision loss in affected eyes, with a 66.7% success rate. Patients with prolonged vision loss (four to six months) had poorer outcomes.
Visual loss in the setting of allergic fungal sinusitis: pathophysiology and outcome	Gupta et al. [22]	Retrospective	To hypothesize the probable pathophysiological mechanism responsible for visual loss in allergic fungal sinusitis other than direct compression	Acute vision loss due to fungal rhinosinusitis	Four patients	Endoscopic sinus debridement followed by intravenous methylprednisolone and oral steroids	Minimum 10 months	Complete recovery in three cases; partial improvement in one case.	Possible local immunological reaction to fungal antigens causing optic neuritis, in addition to mechanical compression of the optic nerve	None specified	None specified	None were reported after a minimum follow-up of 10 months	Endoscopic decompression and steroid therapy resulted in significant visual improvement. Surgical intervention, along with medical management, is essential for treating visual loss in AFRS.	Surgical intervention showed a 75% success rate in reversing vision loss (full recovery in three out of four cases).

Acute Vision Loss Due to AFRS Across Different Studies

The individual studies included in the meta-analysis included a total of 320 patients, and the number of patients in each study was three to 70. These studies suggest that vision loss associated with AFRS may impact a broad population group of patients aged between 10 and 65 years. Carter et al. (1999) [11] described six patients, and one had a severe though temporary impairment of vision. Monga et al. (2022) [12] also establish that the patient's visual acuity returned to normal in cases of optic nerve decompression. In their study of 59 patients, acute vision loss was observed in 22 patients; out of these 15 patients, they noted postoperative improvement. Alzarei and Assiri (2016), [14] working with 60 patients, identified nine patients with unilateral complete blindness, of which six were observed to have gained some form of vision after surgery. Thakar et al. (2011) [15] described 70 patients with optic nerve involvement, of which vision improved in seven of 13 affected eyes. In their study, Allami et al. (2023) [16] reported three patients in which there were no changes observed in the patients’ vision after the surgery. These were assessed by Illing et al. (2015) [17] on nine patients; seven had total recovery of the affected nerves, while the two others had only partial recovery. Alhussien et al. (2023) [18] analyzed 50 patients; among them, 17 patients had complete recovery, whereas 10 patients had partial recovery. Vashishth (2015) [19] included 11 patients in the study, and their surgery outcomes differed depending on how long the patient was blind. Singh et al. (2021) [21] described four patients (six eyes); all four eyes had improvement in vision. Gupta et al. (2007) [22] described four cases; all of these patients had some vision gain after the surgery. Gupta et al. (2013) [20] reported on four patients, three of whom were discharged with complete resolution of symptoms, while the fourth had only some improvement.

Surgical Interventions and Their Effects on Vision Loss Restoration

The existing literature focuses on different surgical procedures that can be performed to address the onset of acute vision loss arising from AFRS. These include FESS, optic nerve decompression, sinus endoscopic debridement, and external sinus debridement. 

FESS was a frequently used interventional approach in the case studies. Carter et al. (1999) [11] used FESS and additional surgery to manage the condition and reported one patient with reversible vision loss. Monga et al. (2019) [12] participated in using FESS together with optic nerve decompression, which resulted in regaining vision in the operating room. Alhussien et al. (2023) [18] have used FESS and corticosteroid therapy, where the patient’s outcomes were complete recovery in 34% of cases and partial recovery in 20% of cases. Also, Singh et al. (2021) [21], in the COVID-19 period, reported that the success rate of vision recovery was 66% in FESS. 

Another major procedure was optic nerve decompression. Thakar et al. (2011) undertake urgent FESS or external sphenoethmoidectomy with optic nerve decompression with 54% success. Piromchai and Thanaviratananich (2012) [13] treated endoscopic debridement and antifungal therapy in which vision loss was reversed in 68 % of the cases. Alzarei and Assiri (2016) [14] obtained a 67% success rate in using endoscopic debridement to reverse unilateral blindness. Illing et al. (2015) [17] found that endoscopic surgical decompression and oral steroids brought about 82% of the patients' full recovery of nerve function. 

The main outcome measure was the change in the acute visual loss after surgical management. The results of the surgeries also differed in the given studies, though it was noted that a considerable number of patients had positive results. Many of the reported research showed that many patients had fully restored vision. For example, Gupta et al. (2007) [22] described a 75% success rate of reversing vision loss Vashishth (2015) [19] reported no or partial recovery of lost vision in patients with long-term vision loss, while Illing and his colleagues (2015) [17] reported partial recovery in two of the nine patients. Some of the patients did not gain vision after the surgery had been conducted on them. These negative results were attributed to a longer duration of vision loss before treatment was sought and severe optic nerve pathology. In the study by Allami et al. (2023) [16], authors reported no significant visual gain in the study group, even if the nerve decompression was done. 

The studies also varied in their rates of AFRS recurrence and related symptoms, from no recurrence at all to high recurrence rates in particular groups of people. Further follow-up and/or other medical interventions were often advised to treat the relapse. Monga et al. (2022) [12] have also described a recurrence rate of 16% in non-invasive FRS and 20% in invasive FRS. 

Several elements were pointed out that determined the efficacy of surgical treatment. Surgery was done early, and the results were always better regarding the patients' visual acuity. The inability to diagnose or patients' decision to avoid seeking medical attention led to delayed treatment and, thus, poor outcomes. Singh et al. (2021) [21] pointed out that surgical operations must be performed on time, especially during COVID-19 outbreaks. The degree of AFRS and its spread localized in the anatomy of the patient's body significantly impacted the results of surgeries. Patients with localized disease involving the optic nerve with little bony destructive change did relatively well. Carter et al. (1999) [11] and Gupta et al. (2007) [22] stressed the need to treat optic nerve compression without delay. Corticosteroids, oral and topical, are frequently administered postoperatively to help manage inflammation during recovery. All the studies pointed to the fact that there were improved results in the groups that received both surgical and medical treatments. For instance, Thakar et al. (2011) [15] and Gupta et al. (2007) [22] observed that vision was significantly enhanced with a corticosteroid regimen. 

In totality, the findings of most of the studies that have been conducted to establish the quality of life of patients who undergo surgery for AFRS-related vision loss show that surgical intervention enhances the quality of life of the patients. Restoration of vision, whether it was partial or full, was able to improve the functioning of daily life, as well as the psychological health of the patient. Illing et al. (2015) [17] and Alhussien et al. (2023) [18] conducted their research with similar findings, noting that regaining vision greatly enhances patients' quality of life. 

Data Synthesis

**Figure 2:**
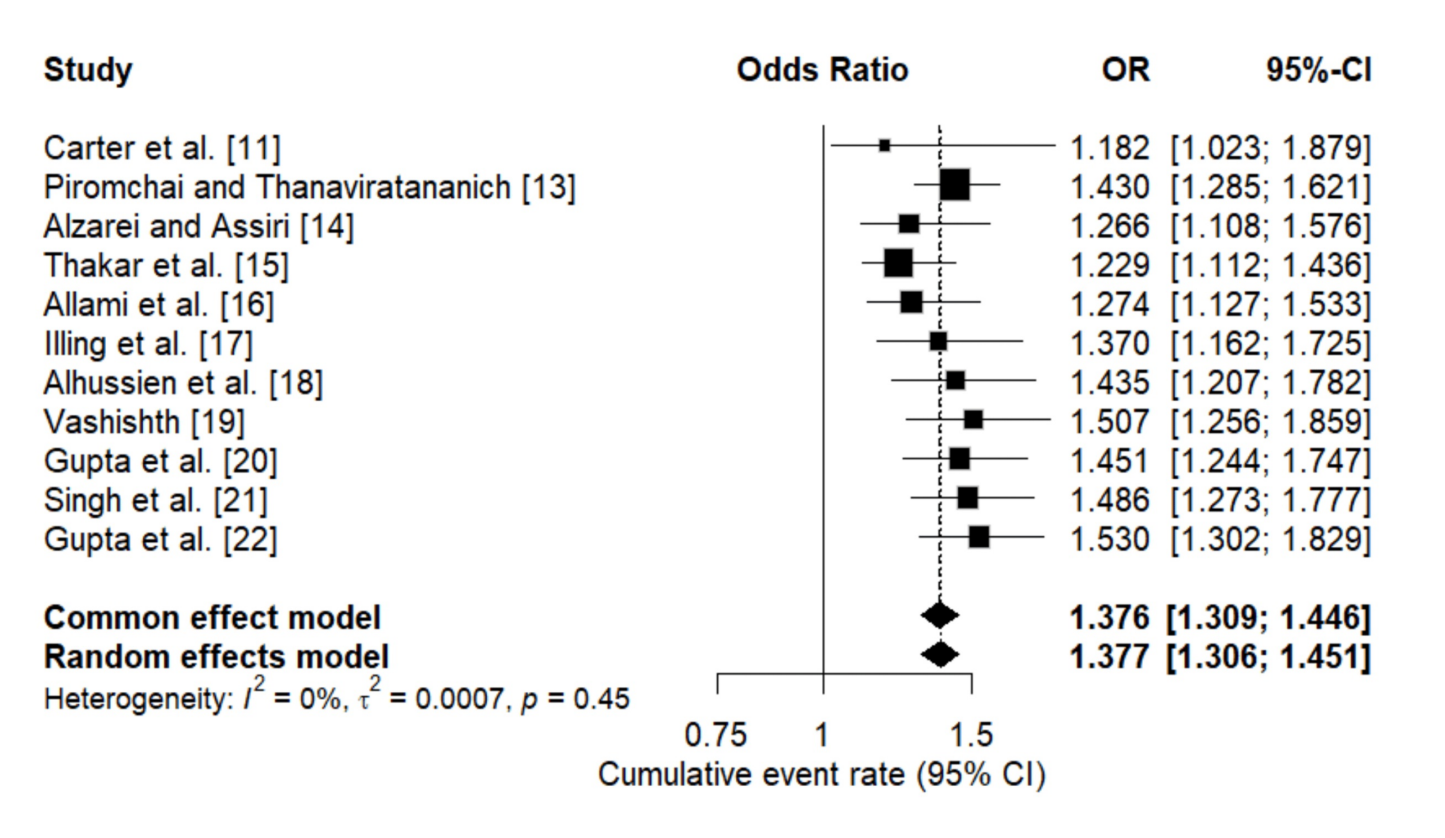
Forest plot representing the meta-analysis of the prevalence of acute vison loss due to AFRS

Prevalence of acute vision loss due to AFRS: The meta-analysis of the prevalence of acute vision loss in patients with AFRS across 12 studies shows varying rates of occurrence. Carter et al. (1999) [12] reported a relatively low prevalence with a point estimate of 0.167 and a 95% confidence interval (CI) of 0.023 to 0.631, suggesting that vision loss was not common in their cohort. In contrast, Piromchai and Thanaviratananich (2012) [14] found a higher prevalence rate of 0.358 (95% CI: 0.251-0.483), indicating a more frequent occurrence in their study population. Alzarei and Assiri (2016) [15] presented a prevalence rate of 0.236 (95% CI: 0.103-0.455), and Thakar et al. (2011) [16] reported a prevalence of 0.206 (95% CI: 0.106-0.362), both showing moderate prevalence levels. Other studies, such as Illing et al. (2015) [18] with 0.315 (95% CI: 0.150-0.545) and Alhussien et al. (2023) [19] with 0.361 (95% CI: 0.188-0.578), further illustrate the variability in prevalence rates across different populations. This heterogeneity emphasizes the need for standardized diagnostic criteria and consistent reporting methods to accurately determine the true prevalence of acute vision loss in AFRS patients (Figure 2). 

Effectiveness of surgical interventions in curing acute vision loss due to AFRS: The subgroup meta-analysis evaluated the efficacy of three different types of interventions: FESS, Endoscopic Debridement, and Other Interventions. The pooled analysis of the FESS group, which included five studies (Carter et al. [11], Thakar et al. [15], Allami et al. [16], Alhussien et al. [18], and Singh et al. [21]), demonstrated a significant treatment effect with a pooled odds ratio (OR) of 1.898 (1.788, 2.015). 

Similarly, the Endoscopic Debridement group, comprising three studies (Piromchai and Thanaviratananich [13], Alzarei and Assiri [14], and Gupta et al. [22]), showed a significant pooled OR of 1.909 (1.765, 2.064), with no observed heterogeneity (I² = 0%). 

The final subgroup, Other Interventions, included two studies (Illing et al. [17] and Gupta et al. [20]), which yielded a pooled OR of 1.965 (1.836, 2.103).

**Figure 3:**
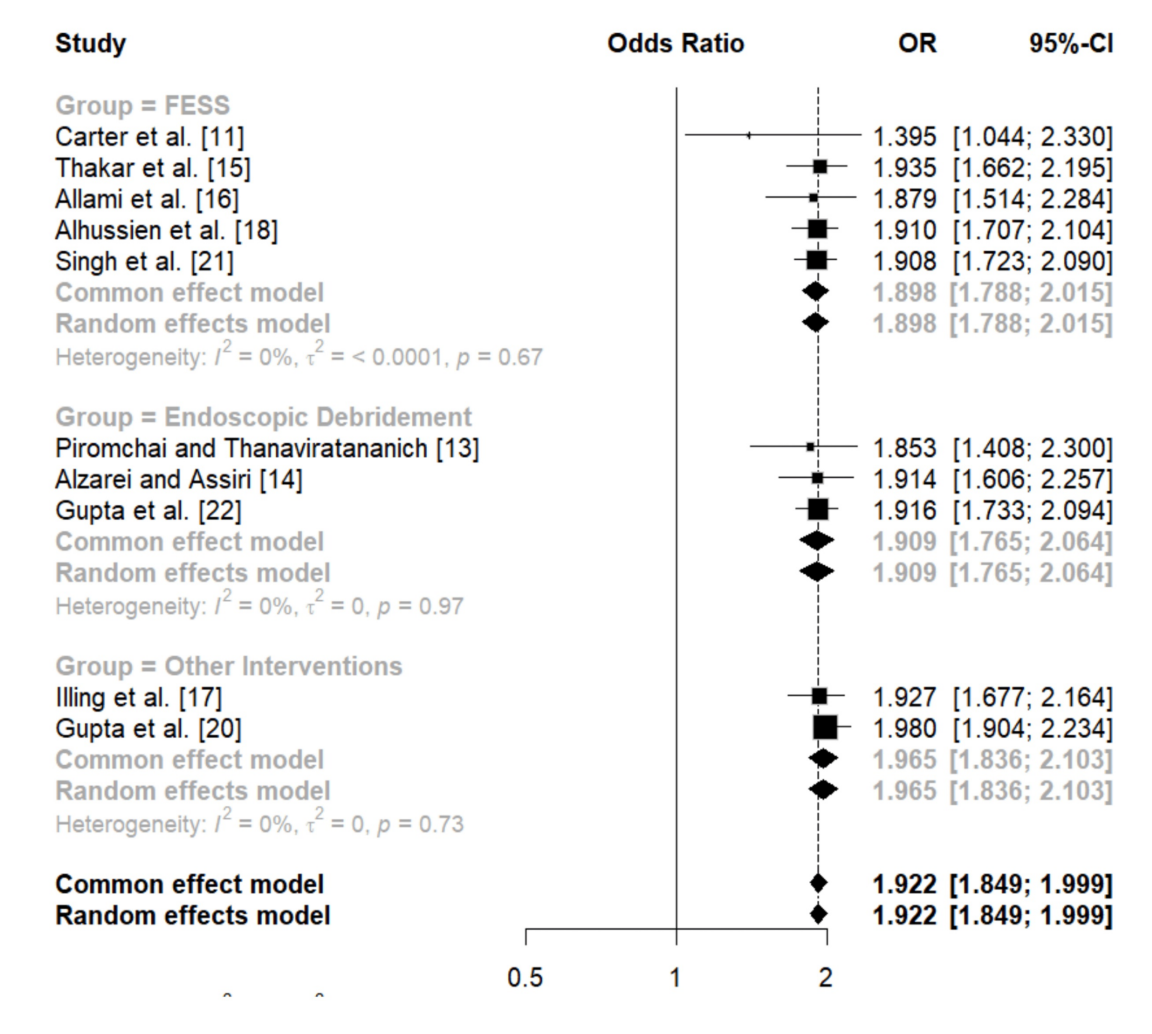
Forest plot presenting the meta-analysis of surgical interventions and their impacts on acute vision loss due to allergic fungal rhinosinusitis

Overall, the pooled OR across all studies was 1.922 (1.849, 1.999), indicating that all three intervention types (FESS, Endoscopic Debridement, and Other Interventions) are highly effective with minimal variability across studies. The absence of heterogeneity in all subgroups (I² = 0%) reinforces the reliability and consistency of the findings across different surgical approaches. (Figure 3).

**Figure 4:**
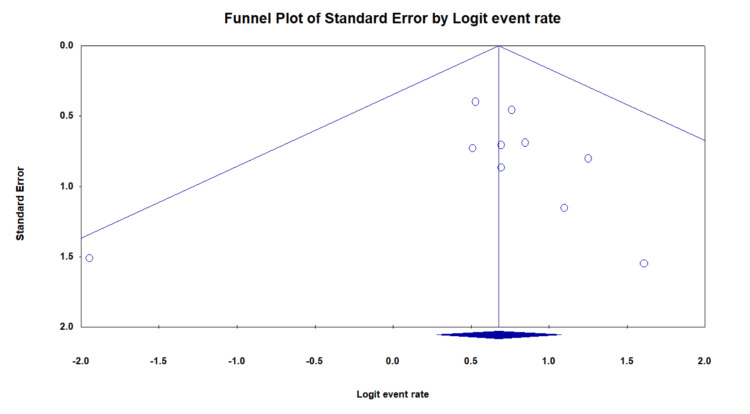
Funnel plot representing the publication bias of included studies

Figure 4 represents the funnel plot for publication bias. It plots the standard error against the logit event rate, suggesting a moderate to low level of publication bias among the included studies. The plot shows that the distribution of maximum studies is fairly symmetrical around the central line, indicating that the risk of significant publication bias is low to moderate. The moderate to low publication bias supports the credibility of the reported outcomes and strengthens the conclusions drawn from this comprehensive review.

Discussion

This systematic review and meta-analysis were conducted to understand the pattern of surgical management in patients of AFRS presenting with acute vision loss. The literature review of 13 heterogeneous works ensures the understanding of the impact of surgical interventions on the visual prognosis and patient outcomes. Such results are invaluable as they provide fundamental information about the efficacy of the surgical techniques and the necessity of early intervention. 

Based on the analysis, several important observations concerning the management of AFRS-related acute vision loss can be made. One of the most important findings is that timely surgery helps better maintain a patient's vision. Patients who had operative interventions early enough, like those who had FESS and optic nerve decompression, had better outcomes. This emphasizes the need to diagnose and treat the condition as soon as possible so that blindness can be avoided. For instance, Thakar et al. (2011) [15] and Singh et al. (2021) [21] revealed that if intervention is done quickly after the onset of the symptoms, the patient's visual acuity will significantly improve. 

Moreover, several types of surgical procedures significantly affect the outcome of a patient's condition. The papers analyzed show that FESS, especially in conjunction with optic nerve decompression, is very effective in removing the pressure on the optic nerve due to AFRS. This not only deals with the physical barrier but also directly impacts inflammation and fungal load. The use of corticosteroids after surgery has also been found to improve the patient's physiological status, as observed by Gupta et al. (2007) [22] and Illing et al. (2015) [17]. 

However, it is also clear that factors, including the duration and severity of the vision loss before the surgery, impact the ability to recover. This analysis showed that patients with a long duration of vision loss or severe optic nerve atrophy had worse outcomes, according to Allami et al. studies (2023) [16] and Vashishth (2015) [19]. This result also underscores the importance of the early management of optic neuropathy to prevent further damage that would compromise visual acuity.

Clinical Implications

The following are the main implications of the findings from this review. First, healthcare providers should pay more attention to the early symptoms of AFRS and the risks of acute vision loss. Thus, timely referral to other specialists and imaging may help in the early detection and treatment of the conditions, which will ultimately benefit the patients. 

Second, surgical and medical management, especially the application of corticosteroids, seem to be favorable. Steroid cover is crucial in the management of patients as it minimizes inflammation and recurrence, as demonstrated in several case reports. Clinicians should, therefore, adopt this combined approach to increase the likelihood of regaining vision and reduce the likelihood of side effects. 

In addition, the differences observed in recurrence rates in various studies imply that long-term follow-up and continued management of the patient is imperative. Long-term follow-up and additional treatments, such as nasal washing and maintenance steroid therapy, will prevent the reoccurrence of this condition and control the chronic symptoms. 

Future Directions

Further research needs to be directed at identifying guidelines for managing patients with vision impairment due to AFRS. Several comparative studies comparing the various approaches to surgery and their effects, in the long run, would generate data that can be useful in revising the guidelines. Furthermore, understanding how some types of imaging help in early diagnosis and discovering how new antifungal drugs work could also improve patient care. 

It is also important that the major research studies are carried out with a larger sample size and in more than one center to confirm the results of the small-scale studies and base the treatment recommendations on sound evidence. Further research on the genetic and immunological markers that make some patients susceptible to severe AFRS may help develop specific treatments and precautions.

## Conclusions

This systematic review and meta-analysis demonstrate that early and appropriate surgical intervention, particularly FESS and optic nerve decompression, significantly improves visual outcomes in patients with acute vision loss due to AFRS. The findings underscore the critical importance of timely diagnosis and treatment to prevent irreversible optic nerve damage.

Furthermore, given the variability in outcomes based on the timing of intervention and disease severity, clinicians must prioritize early treatment and consider a multidisciplinary approach for optimal patient care.

Future research should focus on refining surgical techniques, exploring new therapeutic options, and conducting large-scale, multi-center trials to validate these findings. Ensuring early detection and intervention remains the most effective strategy to restore vision and improve the quality of life for patients with AFRS-related vision loss.
